# Acute inactivation of the serine-threonine kinase Stk25 disrupts neuronal migration

**DOI:** 10.1186/1749-8104-8-21

**Published:** 2013-11-13

**Authors:** Tohru Matsuki, Jianhua Chen, Brian W Howell

**Affiliations:** 1 Department of Neuroscience and Physiology, SUNY Upstate Medical University, 750 E. Adams St, Syracuse, NY 13210, USA; 2 Current address: Institute for Developmental Research, 713-8 Kamiya, Kasugai, Aichi 480-0392, Japan

**Keywords:** Neuronal migration, Brain development, Reelin, Dab1, Sok1, Ysk1, LYK5

## Abstract

**Background:**

Neuronal migration involves the directional migration of immature neurons. During much of the migration period these neurons are polarized with defined leading and trailing processes. Stk25 has been shown to bind to the LKB1 activator STRAD and regulate neuronal polarization and dendritogenesis in an opposing manner to Reelin-Dab1 signaling. It is not known, however, whether Stk25 controls neuronal migration, a key developmental process regulated by Reelin-Dab1 signal transduction.

**Findings:**

Here we find that while constitutive Stk25 deficiency does not lead to neuronal phenotypes, acute reduction by either Cre-mediated gene inactivation or by knockdown causes a developmental neuronal migration error. Furthermore, we find that knockdown of LKB1, STRAD and GM130, molecules that have previously been implicated with Stk25, causes similar aberrations in neuronal migration.

**Conclusions:**

Loss of *Stk25* function early in development likely leads to functional compensation for its roles in neuronal development. Stk25 regulates neuronal positioning, possibly as part of the LKB1-STRAD-Stk25-GM130 pathway that was previously shown to be important for neuronal polarization.

## Findings

### Introduction

The STE20 family serine/threonine kinase Stk25 (also Ysk1, Sok1) has been shown to regulate hippocampal neuronal polarity and dendritogenesis in an opposing fashion to the Reelin-Dab1 signaling pathway. Stk25 participates in cell polarity by interacting with STRADα (also LYK5), an activator of the serine-threonine kinase LKB1. LKB1 is an evolutionarily conserved Par4 homolog central to cell polarity regulation in many cell types. A prior study has shown that Stk25 knockdown hinders LKB1-STRADα-regulated epithelial cell polarization whereas its overexpression restores polarity defects observed in LKB1 knockdown neurons [1]. Furthermore, Stk25 opposes the action of the Reelin-Dab1 signaling pathway to regulate dendritogenesis and neuronal polarization. The Reelin-Dab1 signaling pathway is best known for its role in orchestrating brain lamination by regulating neuronal migration and cell positioning [2]. The opposing relationship between Reelin-Dab1 and LKB1-STRAD-Stk25-GM130 signaling raises the possibility that inactivating the latter may also disrupt neuronal migration. Consistent with this hypothesis, inactivation of the *STRADα* gene disrupts the development of neuronal laminae [3,4], indicating an important role in neuronal migration for this polarity signaling system.

The Reelin-Dab1 signaling pathway was identified through the analysis of mouse mutants with very similar phenotypes. The *dab1* mutant mice and the *Reeler* mutant, which fails to make or secrete the Reelin protein, have very similar lamination defects in the neocortex, hippocampus and cerebellum, among other regions of the central nervous system [5-8]. Mice with compound homozygous mutations in the partially redundant receptor genes *ApoER2* and *VLDLR* also share this phenotype [9]. It was subsequently shown that the Reelin ligand binds to the extracellular domains of ApoER2 and VLDLR and clusters them, leading to the tyrosine phosphorylation of Dab1 [10,11]. Tyrosine-phosphorylated Dab1 acts to nucleate signaling complexes, particularly with the adaptor proteins Crk and CrkL that ultimately regulate cell surface cadherin expression [12-15]. Consistent with a role for cadherins on the pathway, dominant negative N-cadherin expression leads to migration defects in embryonic neurons, and N-cadherin overexpression partially rescues defects caused by *dab1* gene disruption [14,15].

STRADα is a pseudokinase that binds to LKB1, recruits it to the cytoplasm, stabilizes it and leads to its activation [16]. LKB1 phosphorylates and activates over a dozen downstream kinases [17], which are involved in cell proliferation, polarization and migration, in addition to energy metabolism [18]. One branch of this pathway that is relevant to neuronal positioning is the AMPK pathway that regulates mTOR signaling [3]. LKB1 activates AMPK, which phosphorylates and activates mTSC2, blocking mTOR function and subsequently inhibiting S6 kinase activation. Patients with homozygous recessive STRADα mutations develop polyhydramnios, megalencephaly, and symptomatic epilepsy (PMSE) [19]. PMSE shares some similarities to tuberous sclerosis caused by inactivating mutations in TSC2, including cortical dysplasia, epilepsy and neurons with abnormal morphology [3].

Stk25 has been shown to regulate polarized migration in cultured cells, and this activity is regulated through its interactions with GM130 [20], a ubiquitously expressed Golgi-shaping protein. GM130 has been shown to regulate tethering of ER-derived vesicles with the Golgi [21,22]. GM130 knockdown, like Stk25 and LKB1 knockdown, causes loss of axonal initiation, the first step of neuronal polarization [1]. The Golgi is believed to play an important role in cell polarization [23-25]. GM130, Stk25 and LKB1 knockdown causes Golgi dispersion [1], which could reduce its ability to contribute to cell polarity. Stk25 overexpression did not rescue the Golgi dispersion or polarity defects caused by GM130 knockdown, possibly suggesting that GM130 acts downstream of Stk25 [1]. The role of this pathway and the Golgi apparatus in neuronal migration is not known.

Here we made use of a novel *Stk25* conditional mutant mouse to both constitutively and acutely inactivate *Stk25* during brain development to assess its role in neuronal positioning. In addition, by *in utero* knockdown, we examined the roles of LKB1, STRADα and GM130 in this process.

### Results

**Figure 1:**
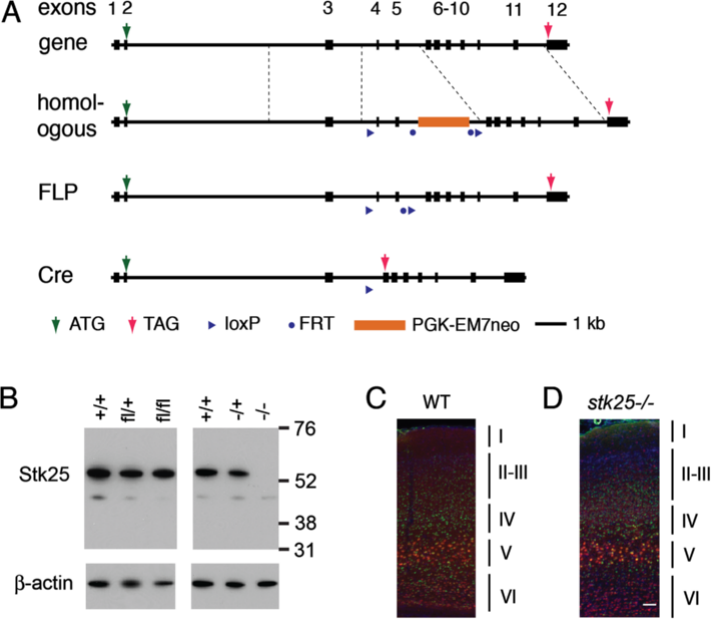
**Generation of *Stk25 *conditional mice. A)** Exons 4 and 5 were floxed by homologous recombination, which introduced a PGK-EM7 neo drug-selectable marker. The Frt-flanked marker was removed by FLP-mediated recombination. Cre-mediated recombination removes exons 4 and 5, resulting in a frameshift and termination in exon 6. The product of homologous recombination (homologous), FLP-mediated recombination (FLP) and Cre-mediated recombination (Cre) are shown. **B)** Stk25 protein levels were measured by western blotting (anti-Stk25; [1]) in brain lysates of P0 mice with the indicated genotype. The *Stk25*^*fl/fl*^ (fl/fl) allele is fully functional for Stk25 expression and the germline expression of Cre generated a null allele with no detectable Stk25 expression in the homozygous state (−/−). **C)** The positions of Satb2-positive layer II-IV and Ctip2-positive layer V-VI neurons are evident in the neocortex of two-month-old wild-type mice. **D)** An indistinguishable pattern of lamination was observed in the *Stk25*^*−/−*^ mice. Cortical layers are indicated to the left of the images (Bar 50 μm).

To determine whether Stk25 has a role in neuronal positioning, we generated a conditional *Stk25* mouse model by floxing exons 4 and 5 (Figure 1A). The floxed allele supported normal Stk25 protein expression (Figure 1B). Cre-mediated excision is predicted to cause a frameshift and translational termination (Figure 1A). As a first step in the analysis, we generated an *Stk25* knockout line by inactivating the *Stk25* gene in the germline by transgenic Cre expression. Western blots of brain lysates from mice that are homozygous for this inactivated *Stk25* allele (*Stk25*^*−/−*^) had no detectable Stk25 protein expression at postnatal day (P) 0, suggesting that this represents a null allele (Figure 1B).

The *Stk25*^*−/−*^ mice were born at the expected Mendelian frequency. These animals were indistinguishable from littermates based on body size or behavior. Since we had previously shown that Stk25 knockdown reduces axon initiation *in vitro* and *in vivo*, we examined the major nerve tracts in adult mice. We found no significant reduction in size of the major nerve bundles by standard histological staining (data not shown). It is possible that with more extensive analysis, subtle changes in nerve tracts might be uncovered. However, the brain histology clearly was at odds with our expectations from our *in utero* knockdown study, in which we found approximately 40 percent of neurons failed to extend an axon [1]. In addition, no significant disruption of layers was revealed by immunostaining for the layer V-VI marker Ctip2, or the layer II- IV marker Satb2 (Figure 1C,D). This was consistent with the histology, which showed no obvious disruption of lamination in the cortex, hippocampus or cerebellum (data not shown).

**Figure 2:**
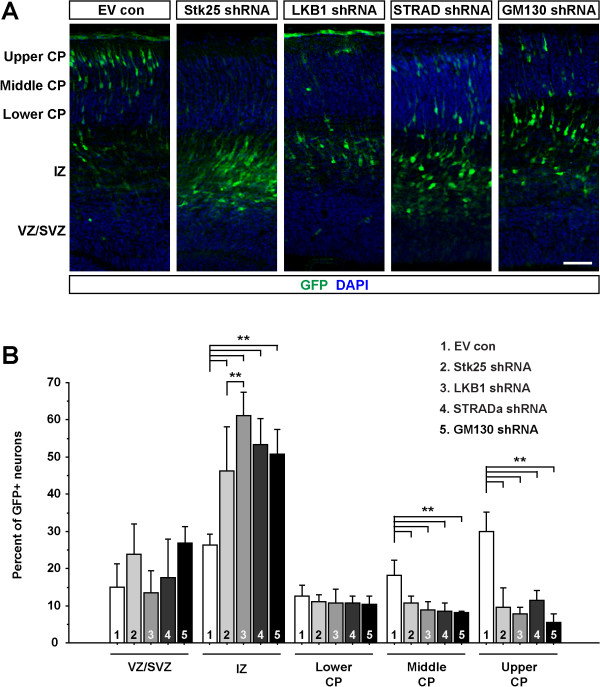
**Knockdown of Stk25 and signaling partners leads to anomalous neuronal migration. A)** Knockdown or control (empty) vectors were electroporated into E14.5 mouse cortex and the position of GFP-tagged neurons were examined at E17.5. **B)** The knockdown of Stk25, LKB1, STRADα or GM130 decreased the number of neurons in the upper cortical plate (CP) and increased the number of neurons held up in the intermediate zone (IZ) as compared to empty vector control (EV) electroporated cells (*P <0.0001 ANOVA with Tukey-Kramer post-hoc test). Ventricular zone/Subventricular zone (VZ/SVZ).

The discrepancy between our previous results based on Stk25 knockdown [1], which indicated a role for Stk25 in neuronal polarization and axon initiation, and the lack of a phenotype in the nerves tracks of *Stk25*^*−/−*^ mice led us to investigate whether there was compensation for the loss of *Stk25* gene function, which has been reported for other knockouts. If this was the case, we reasoned that acute loss of Stk25 might cause a neuronal positioning phenotype. To examine this we knocked down Stk25 expression *in utero* by electroporating a construct that expresses a previously validated shRNA [1] at embryonic day (E) 14.5. The position of GFP-labeled neurons at E17.5 was compared between brains electroporated with the knockdown and an empty vector (Figure 2A,B). In the Stk25 knockdown brains a high percentage of neurons stalled in the intermediate zone, whereas in the empty vector (EV) control they migrated into the cortical plate.

The above results are consistent with the hypothesis that the inactivation of *Stk25* needs to be acute in order to observe a neuronal phenotype. However, there are other possibilities that could explain the discrepancies between the knockdown and knockout phenotypes. For instance, perhaps the predicted residual 87 N-terminal Stk25 peptide generated from the exon 4 to 5-deleted *Stk25* gene sustains some function. It is also formally possible that the phenotype is revealed only during a developmental window and not observed in adults. To test these possibilities, we asked if acute inactivation of homozygous floxed *Stk25* (*Stk25*^*fl/fl*^) would produce a similar phenotype to Stk25 knockdown and whether analyzing the migration of *Stk25*^*−/−*^ neurons in the E14.5 to E17.5 time frame would reveal differences from wild-type.

**Figure 3:**
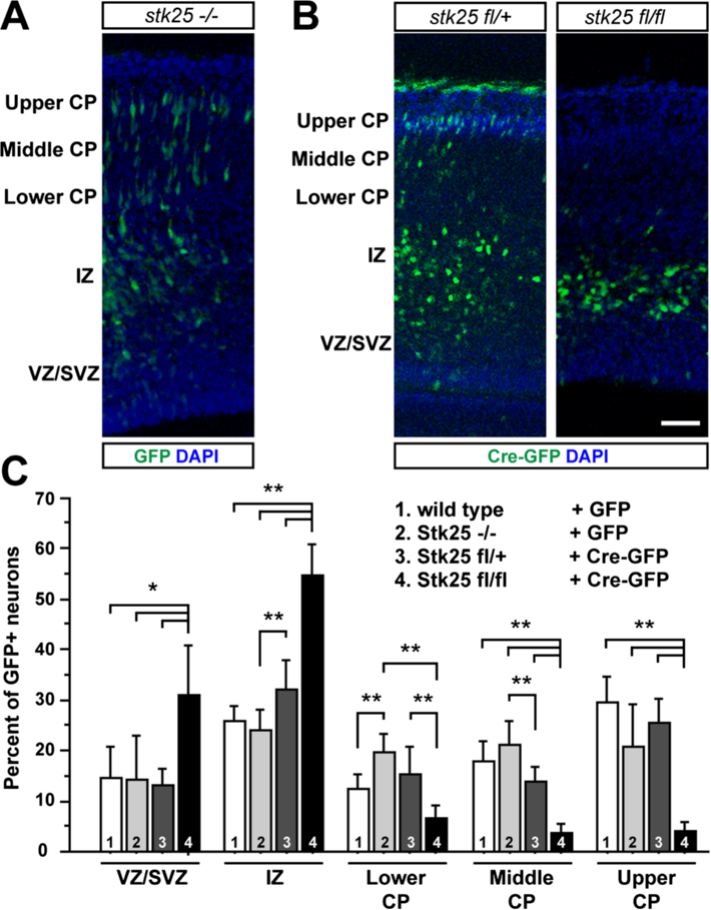
**Acute inactivation of the conditional *Stk25 *allele by Cre causes a neuronal migration anomaly. A)** The electroporation of GFP-encoding DNA into the developing cortex of E14.5 embryos marks neurons that have entered the upper portion of the cortical plate (CP) by E17.5. **B)** In contrast, *Stk25*^*fl/fl*^ neurons that express GFP-Cre fail to populate the cortical plate and instead many are located in the intermediate zone. GFP-Cre-expressing neurons in the *Stk25*^*fl/+*^ mice migrate in a fashion similar to wild-type GFP-expressing neurons. **C)** Quantification of the GFP-expressing neurons in the indicated regions of the neocortex demonstrates that *Stk25*^*fl/fl*^ neurons exposed to Cre (4) are statistically more likely to be in the intermediate (IZ) or subventricular (SVZ) zones and less likely to be in the cortical plate than *Stk25*^*fl/+*^*;* Cre-GFP (3), *Stk25*^*−/−*^*;* GFP (2) or wild-type; GFP (1) neurons (*P = 0.001, **P <0.0001 ANOVA with Tukey-Kramer post-hoc test).

Interestingly, the GFP-labeled neurons in *Stk25*^*fl/fl*^ mice that were acutely exposed to Cre expression stalled their migration in the intermediate zone (Figure 3B). In contrast, expression of CRE-GFP in *Stk25*^*fl/+*^ embryonic neurons did not cause an arrest in neuronal migration. These neurons migrated similarly to wild-type neurons expressing GFP (Figure 3B,C). This suggests that expression of the CRE-GFP fusion protein does not cause erroneous migration and that half the normal levels of Stk25 are sufficient to sustain neuronal migration. Similarly *Stk25*^*−/−*^ neurons labeled with GFP migrated normally (Figure 3A,C). This shows that there was a distinct difference in neuronal migration between animals where the *Stk25* gene was inactivated from the time of conception and those where Stk25 was inactivated acutely at E14.5 in a subset of neurons.

Stk25 has previously been linked to STRADα and GM130 through protein-protein interactions. In addition, Stk25 was shown to partially rescue Golgi fragmentation and loss of axon initiation defects observed in LKB1 knockdown neurons [1]. We thus investigated whether LKB1, STRADα or GM130 plays a similar role in neuronal positioning by knocking down these molecules at E14.5 with vectors expressing the respective shRNAs and co-expressing GFP. The position of GFP- labeled neurons was analyzed in the brains of E17.5 pups.

The knockdown of LKB1, STRADα and GM130 reduced the number of GFP- tagged neurons in the cortical plate and increased the number that were observed in the intermediate zone, albeit to different extents. Interestingly LKB1 knockdown caused the greatest increase in neurons in the intermediate zone, though the brain-specific knockout of this gene did not cause an apparent neuronal positioning error [26]. Our findings with STRADα are consistent with other studies that have shown a reduction in the number of correctly positioned neurons. The finding that GM130 is required for neuronal positioning is the first evidence to our knowledge that expression of a Golgi protein is involved in neuronal migration.

### Discussion

In this study, we demonstrate that Stk25 and molecules with a physical or functional link to Stk25 have a role in neuronal positioning during brain development. We determined that acute knockdown of Stk25 by shRNA produced a migration disruption, which was surprising given the lack of neuronal position phenotype in adult *Stk25*^*−/−*^ mutant mice. Furthermore, we found that the acute inactivation of the *Stk25* gene at E14.5 by Cre-mediated excision led to aberrations in neuronal positioning that are apparent by E17.5. In contrast, the GFP-labeled neurons in the *Stk25*^*−/−*^ mice did not have an obvious positional phenotype in the developing brain. This suggests that when *Stk25* is inactivated for an extended period prior to neuronal exit from the proliferative zone there is some form of compensation. We confirmed results that knockdown of STRADα and LKB1 caused erroneous migration [4,27] and demonstrate that this is qualitatively similar to the defects caused by Stk25 deficiency (Figure 2A,B). We also find similar migration phenotypes when the Golgi-organizing protein GM130 is knocked down (Figure 2A,B). This suggests that in addition to working together during neuronal polarization as previously shown [1], the gene products Stk25, LKB1, STRADα and GM130 also act on a common pathway to regulate neuronal positioning.

Discrepancies between the knockdown and the knockout phenotypes have been observed for several genes including: *dcx*[28,29], *dclk*[30,31], *APP*[32,33], and *PKCδ*[34,35]. These discrepancies have been attributed to compensation for the loss-of-gene function; however, this has not been experimentally tested. Here we show that depending on the mechanism of inactivation of the *Stk25* conditional allele, we either observed no phenotype or a dramatic phenotype (compare Figure 3B with Figure 3A). The acute inactivation of the *Stk25* gene by Cre-mediated recombination led to an accumulation of neurons in the intermediate zone (Figure 3B,C), very similar to the *Stk25* knockdown phenotype (Figure 2A,B). In contrast, tagging migrating neurons in constitutive *Stk25*^*−/−*^ mice with GFP did not reveal a neuronal migration disruption (Figure 3A,C). Furthermore adult *Stk25*^*−/−*^ mutants had normal brain histology (Figure 1D). Thus, only when the *Stk25* gene is inactivated acutely is a role in neuronal migration apparent. It remains to be determined how the cells compensate for *Stk25* loss-of-function; for instance, are related genes upregulated or is compensation more complex, involving the different effectors of Stk25 signaling?

Another difference between the acute and the constitutive inactivation of Stk25 is that the acute inactivation was limited to a subset of neurons surrounded by wild-type cells; the knockout eliminated Stk25 expression in every cell. It is possible that Stk25-null cells, in a null environment have a competitive advantage for migration compared to mutant cells in a wild-type environment. However this seems unlikely.

Inactivation of the Reelin-Dab1 pathway also causes neuronal positioning errors that have been documented in knockout and RNA interference experiments [36]. Inactivation of *dab1* at E16 causes a failure to enter the cortical plate at E20 and E21 when control cells are concentrated in this region. The similarity of the neuronal migration phenotypes, however, should not be seen as evidence that Stk25 and Reelin-Dab1 signaling have synergistic effects. Similarities in neuronal positioning effects have also been observed for knockdown of MARK2/par-1 and DCX, which have the opposite effect on microtubule stability [37]. The inactivation of either causes neuronal migration failures, but the combination of the mutations partially rescues the migration phenotypes. It remains to be determined if inactivation of the genes encoding the players of the Reelin-Dab1 pathway and Stk25 simultaneously would increase or decrease the severity of the observed migration defect.

STRADα is thought to regulate neuronal positioning through mTor regulation [4,19]. Accordingly, the AMPK branch of the pathway phosphorylates and inhibits TSC2 leading to mTOR suppression and reduced activity of S6 kinase and decreased phospho-S6. Consistent with this model, both PMSE and tuberous sclerosis patients have elevated levels of phospho-S6. Similarly, STRADα deficiency led to an aberrant increase in phosph-S6 in cultured neural progenitor cells [4]. Together, this suggests that failure to inactivate mTor is one of the underlying biochemical defects in the neurons with inactivating mutations in STRADα.

The evidence for other molecules on the pathway is not as straightforward, however. The brain-specific knockout of LKB1 has no apparent lamination defect despite very obvious defects in axonal production [26], and knockdown of LKB1 at E18 did not cause neuronal migration defects [38]. In contrast, both a previous study and ours demonstrate neuronal positioning errors in LKB1-deficient neurons by *in utero* knockdown at E14 [27]. Perplexingly, a brain-specific deletion of the genes encoding the two catalytic subunits of AMPK (AMPKα1 and AMPKα2) had no noticeable effects on neuronal migration [39]. Thus, work is required to determine the interplay between downstream effectors of the LKB1 signaling pathway and their effects on neuronal positioning. Intriguingly, mTor is predicted to be upregulated by Reelin-Dab1 signaling through AKT suppression of TSC2 [40,41]. It may therefore represent a point of convergence of Reelin-Dab1 and Stk25 signaling.

Another downstream target of these pathways that may be involved in regulating neuronal positioning is GM130. This study represents the first evidence to our knowledge that a Golgi protein is involved in regulating neuronal migration. Work remains to determine how the Golgi may participate in the process. It has previously been shown that the local positioning of the Golgi or Golgi outposts influences Golgi growth and branching [42,43]. It has been postulated that dendritic branching may influence neuronal positioning by facilitating neuronal anchoring to a particular lamina [44]. The position of the centrosome/Golgi complex also has important roles in neuronal polarity regulation. Neuronal morphology, polarity and Golgi/centrosome position have been shown to be dynamic during neuronal migration [45,46]. Thus alterations in the Golgi, which hinder the normal dynamic transport of cargo proteins, may perturb the migratory path of the migrating neuron.

### Conclusions

Compensation for developmentally early *Stk25* loss-of-function seems likely since we observe dramatic phenotypes when the loss was acute in neuronal progenitors at E14.5. Examining the null animals, which have the same Cre-mediated gene rearrangement, with the same method of *in utero* labeling of neurons did not show any detectable position anomaly. Neither were there any lamination or nerve tract defects observed in the *Stk25*^*−/−*^ adults, which would be expected from the migration anomalies and the loss of axon initiation observed by acute *Stk25* knockout or Stk25 knockdown (Figure 1; [1]). Given the previously documented physical interactions between Stk25, GM130 and STRAD and the similarities of the neuronal positioning phenotypes of the Stk25, LKB1, STRAD, and GM130 knockdowns, it seems likely they act on the same pathway to regulate neuronal migration. Further studies are required to resolve the interplay between the Reelin-Dab1 and Stk25 pathways to determine how they interface to regulate neuronal positioning during development.

### Methods

#### Generation of the Stk25 floxed and knockout mice

Exons 4 and 5 of the *Stk25* gene were floxed and a PGK-EM7 neomycin cassette flanked by Frt sites was introduced into intron 5 by recombineering a BAC to generate the knockout vector (Figure 1A). The vector was electroporated into C57BL/6-129/Sv hybrid ES cells at the University of Connecticut transgenic core (Farmington CT, USA). Cells that had undergone homologous recombination were identified by PCR. Recombinant ES cells were injected into blastocysts that were then transplanted by standard techniques into pseudopregnant dams. The PGK-EM7 neomycin resistance cassette was removed by breeding the chimeric founder mice with Flp-expressing transgenic mice leaving sole Frt and loxP sites in the intron after recombination (Figure 1A). The Flp transgene was outbred by backcrossing with wild-type C57BL/6 mice. The resultant animals are referred to here as heterozygous or homozygous floxed *Stk25* (*Stk25*^*fl/+*^ or *Stk25*^*fl/fl*^, respectively) mice. Cre-mediated excision of exons 4 and 5 is predicted to cause the frameshift-termination of translation in exon 6 after translating 87 residues of the Stk25 N-terminus (Figure 1A).

Heterozygous *Stk25* knockout (*Stk25*^*−/+*^) mice were generated by breeding *Stk25*^*fl/+*^ and the transgeneic germ-line-deleter, Meox2-Cre (B6.129S4-Meox2 < tm (Cre)Sor; Jackson Labs, Bar Harbor ME, USA) mice. Progeny that were positive for the *Stk25* gene modification were backcrossed with C57BL/6 to outbreed the Meox2-Cre transgene.

To distinguish the conditional allele from the wild-type, the following primers that flank the 5′ loxP site were used: P1 (tcagagaggccttttctcca) and P2 (gccagcctggtctacagatt). The PCR products were 293 bp for the wild-type allele and 375 bp for the mutant. To identify the allele generated by the excision of exons 4 and 5, P1 was used in conjunction with primers that flank the Frt site, P3 (cccatttttaatggccacac) and P4 (gggggacctacaggaacatt). The wild-type and mutant alleles produce 468 and 345 bp products, respectively.

Mice used for this manuscript were either on a mixed C57BL/6-129/SV (backcrossed to C57BL/6 at least four times) or Swiss Webster backgrounds. For *in utero* electroporation with Cre (*Stk25*^*fl/+*^, *Stk25*^*fl/fl*^; Figure 3B,C) the mice were C57BL/6-129/SV. The null allele was backcrossed four times onto the Swiss Webster background for histological analysis (Figure 1C, data not shown) and *in utero* electroporation with GFP (*Stk25*^*−/−*^, wt; Figure 3A,C). Finally wild-type Swiss Webster mice were used for *in utero* electroporations with shRNAs (Figure 2A).

All mice were treated ethically and humanly according to National Institutes of Health (NIH) care and use guidelines following protocol (#267), which was approved by the Committee for the Humane Use of Animals at SUNY Upstate Medical School.

Heterozygous *Stk25* knockout (*Stk25*^*−/+*^) mice were generated by breeding *Stk25*^*fl/+*^ and the transgenic germline-deleter, Meox2-Cre (B6.129S4-Meox2 < tm (Cre)Sor; Jackson Labs) mice. Progeny that were positive for the *Stk25* gene modification were backcrossed with C57BL/6 to outbreed the Meox2-Cre transgene.

#### DNA constructs

For the *in utero* electroporations a modified version of pLL3.7 was used that has a CMV enhancer/chicken β-actin promoter-driving GFP and the U6-promoter driving shRNA synthesis (pLC) [1]. The shRNAs used to knock down expression of Stk25, LKB1, and GM130 have been described previously [1]. The shRNA to knock down STRADα was generated to the target sequence 5′-gcagcaaccttagcatgatta-3′ (NM028126; bases 533–553). The STRADα shRNA reduced by over 80% the expression of Myc-tagged murine STRADα in HEK293T cells (data not shown).

The pCAG-Cre: GFP vector was obtained from Addgene (http://www.addgene.org) and was initially constructed by the Cepko lab [47].

#### Histological analysis

For immunohistochemistry, brains were perfused with 4% paraformaldehyde in Pagano solution (250 mM sucrose, 25 mM 4-(2-hydroxyethyl)-1-piperazineethanesulfonic acid pH 7.4, 2.5 mM Magnesium acetate, 2.5 mM KCl), post-fixed overnight and then cryopreserved in 40% sucrose in PBS for two days. The brains were sectioned (40 μm thick) using a cryostat (CM1900; Leica Biosystems, http://www.leicabiosystems.com). Sections were immunostained in 3% BSA and 1% ovalbumin in Tris-buffered saline (TBS) containing 0.5% Triton X-100 with anti-Ctip2 (rat IgG, Abcam, Cambridge MA, USA) and anti-Satb2 (mouse IgG, SatbA4B10; Abcam) overnight followed by anti-rat IgG and anti-mouse immunodetection (Alexa 568, Alexa 488, respectively; Invitrogen, http://www.lifetechnologies.com/us/en/home/brands/invitrogen.html). Slides were mounted with Vectashield (Vector Labs, Burlingame CA, USA) containing DAPI (Sigma, St. Louis MO, USA). Brain sections were magnified with a 10X objective and images were collected by fluorescence microscopy (ImagerA2, Zeiss, Oberkochen, Germany) with a digital black and white CCD camera (QImaging, Redwood City CA, USA). Several sections were collected for each brain slice and these were stitched together using NIS-elements software (Nikon, Tokyo, Japan). Images were pseudocolored and intensities adjusted using Photoshop software (Adobe, San Jose CA, USA).

For histological staining (not shown), brains were fixed in 70% ethanol, dehydrated with 100% ethanol, clarified with xylenes and imbedded in paraffin. Coronal sections were taken throughout three brains of each genotype (wild-type, *Stk25*^*−/+*^ and *Stk25*^*−/−*^) every 10 μm and stained with hematoxylin and eosin for analysis.

Electroporated embryos were perfused at E17.5 with 4% paraformaldehyde in Pagano solution and post-fixed for 2 to 3 h. Brains were then embedded in 10% porcine gelatin (Sigma, St. Louis MO, USA) and post-fixed in 4% paraformaldehyde/Pagano’s for 24 h. Brains were sectioned on a VT100S vibratome (50 μm thick; Leica Biosystems, http://www.leicabiosystems.com). GFP- positive sections were collected, immuno-stained with chicken anti-GFP antibody (Invitrogen) and mounted on slides with DAPI containing Vectashield (Vector Lab, Burlingame CA, USA) for visualization. Bin counts for GFP-positive cells were collected from three sections from each of three independent electroporated brains for the indicated samples.

#### Western blotting

Lysates were prepared from P0 mouse brains of the designated genotypes in RIPA buffer (20 mM Tris–HCl (pH 7.4), 150 mM NaCl, 1% Nonidet P40, 2 mM EDTA, 1% sodium deoxycholate, 0.1% SDS, 5 mM 2-mercaptoethanol, 50 mM sodium fluoride, phosphatase inhibitor cocktail 1 (Sigma, St. Louis MO, USA), 1 mM phenylarsine oxide (Sigma, St. Louis MO, USA) and protease inhibitors (complete mini EDTA-free; Roche Applied Science, Penzberg, Germany). Samples were sonicated for 5 seconds with a probe sonicator to shear DNA and stored on ice for 10 min to extract proteins. Samples were clarified by centrifugation at 20,000 × g to remove insoluble material. Samples were normalized for total protein content using a bicinchoninic acid (BCA) kit (Pierce, Rockford IL, USA). Equal amounts of protein mixed with twice concentrated sample buffer (4% SDS, 20% sucrose, 10% glycerol, 125 mM Tris–HCl pH 6.8, 100 mM dithiothreitol, and 0.02% bromophenol blue) were resolved on NuPAGE™ Novex 4-12% Bis-Tris polyacrylamide gels (Invitrogen, http://www.lifetechnologies.com/us/en/home/brands/invitrogen.html), transferred to Immobilon P (Millipore, Billerica MA, USA) and immunoblotted to detect endogenous Stk25 (anti-Stk25 [1]; Santa Cruz Biotechnology, Santa Cruz CA, USA) essentially as described previously [48].

#### In utero electroporations

In utero electroporations were performed on E14.5 embryos as described in previous studies with minor modifications [26,36]. To introduce indicated DNA vectors into neuronal progenitors located in the cortical ventricular zone of indicated genotype of mice, DNA vectors (1 μl of 1 mg/mL, 0.01% FastGreen (Sigma, St. Louis MO, USA) in PBS) were injected into the lateral ventricles of E14.5 embryos in the uteri of deeply anesthetized dams [36]. Tweezertrodes were placed on either side of the uterus at the level of the injected DNA and four 100 msec square pluses of 40 mV current separated by 100 msec intervals were applied to the head of the embryo using an ECM 830 electroporator (BTX, Harvard Apparatus, Holliston MA, USA). GFP-positive embryos were perfused at E17.5 and prepared for histological analysis.

## Abbreviations

EV: Empty vector; PBS: Phosphate buffered saline; PMSE: Polyhydramnios, megalencephaly and symptomatic epilepsy; Stk25fl/+: Heterozygous floxed *Stk25*; Stk25 −/+: Heterozygous for the inactivated *Stk25* allele; Stk25fl/fl: Homozygous floxed *Stk25*; Stk25−/−: Homozygous for the inactivated *Stk25* allele; SDS: Sodium dodecyl sulfate; Tris: Tris(hydroxymethyl)aminomethane; DAPI: 4′,6-diamidino-2-phenylindole.

## Competing interests

The authors declare that they have no competing interests.

## Authors’ contributions

TM collected the data and analyzed the results for Figures 1B, 2 and 3, BH and JC collected the data for Figure 1C, D and histological staining (not shown). The manuscript was written by BH with assistance from TM. All authors read and approved the final manuscript.
